# Effectiveness of spinal cord stimulation in diabetic patients with chronic limb-threatening ischemia: small cohort study

**DOI:** 10.3389/fsurg.2024.1451622

**Published:** 2024-12-09

**Authors:** Anna E. Cyrek, Dietrich Koch, Arkadius Pacha, Sonia Radunz

**Affiliations:** ^1^ Department of General, Visceral, Vascular and Transplant Surgery, University Hospital Essen, University Duisburg-Essen, Essen, Germany; ^2^ Institute of Pharmacology and Toxicology, Ruhr-University Bochum, Bochum, Germany

**Keywords:** critical limb-threatening ischemia, peripheral arterial disease, neuromodulation, long-term results, limb salvage, quality of life, diabetic patients

## Abstract

**Background:**

Chronic limb-threatening ischemia (CLTI) is the most severe form of peripheral artery disease (PAD). Patients with diabetes mellitus (DM) have a faster progression of PAD and a fourfold increased risk of CLTI compared to patients without DM. Epidural spinal cord stimulation (SCS) has been used as a method to improve microcirculation, relieve ischemic pain and reduce the number of amputations in patients with PAD. This is a retrospective small cohort analysis of patients with diabetes and the long-term treatment effect of spinal cord stimulation.

**Methods:**

As the main outcome of the study, we evaluated the survival and amputation of 13 diabetic patients with chronic lower-limb ischemia who were not eligible for surgical or interventional therapy. Secondary outcomes included ankle-brachial index (ABI), ischemic pain intensity, quality of life, use of analgesic medications and skin wound outcomes analyzed during long-term follow-up.

**Results:**

Between January 2010 and January 2017, 13 patients underwent SCS implantation in our vascular center. At 1-year follow-up, the limb salvage rate was 92.3% (12 of 13 patients), and limb ulcers healed in 75% of patients (6/8). No patient died during the one-year follow-up. A total of 4 of patients (31%) experienced major amputation during long-term follow-up, all of them were Fontaine stage IV. Pain intensity and quality of life improved significantly at 6-month follow-up (*p* < 0.05). ABI measurements were unaffected by SCS treatment. There were no complications related to the procedure or device.

**Conclusions:**

SCS is a promising treatment option for diabetic patients unsuitable for endovascular or surgical revascularization. The method improves limb survival in diabetic patients with critical limb ischemia, provides significant pain control, and improves patients' quality of life. However, more studies are needed to clarify the indications for SCS and clarify its effects on the vascular system.

## 1 Introduction

The most severe form of peripheral artery disease (PAD) is chronic limb-threatening ischemia (CLTI) (1). In patients with CLTI who are not candidates for revascularization, the most common six-month amputation rates are 10%–40% and 1-year mortality is 25% (1–3). In patients with diabetes (DM), PAD progresses more rapidly (4) and the risk of CLTI is four times higher than in patients without DM (2). In addition, CLTI patients with DM have a worse prognosis because they are usually old, frail and at high risk for cardiovascular events (2).

Up to 50% of CLTI patients are ineligible for first-line treatment such as bypass surgery or endovascular interventions (5). The non-option status of these patients is due to extensive and diffuse, often infrapopliteal, atherosclerotic lesions, comorbidities and/or lack of an adequate bypass graft (6). Various revascularization techniques have been used to salvage patients with CLTI who are not candidates for bypass or endovascular intervention. However, the effectiveness of these interventions remains unclear (7–10).

Spinal cord stimulation (SCS) is a neuromodulatory technique that represents an alternative treatment for patients with CLTI who are unsuitable for vascular reconstruction procedures. In previous studies, SCS has been shown to reduce pain, avoid or delay amputation, and improve quality of life (11–14).

This is a retrospective small cohort study analysis of the long-term therapeutic effect (limb salvage, wound closure, and clinical changes) of spinal cord stimulation in diabetic patients.

## 2 Materials and methods

### 2.1 Study population

This retrospective, single-center small cohort analysis of spinal cord stimulation procedures included patients with diabetes from January 2010 to January 2017. All study methods followed the Declaration of Helsinki and were approved by the Institutional Ethics Committee of the Medical Faculty of the University of Duisburg. Essen (20-9609-BO). All patients gave written informed consent for SCS implantation.

Study inclusion criteria were age > 18 years, CLTI (Fontaine grade III-IV) in diabetics, ankle-brachial index (ABI) < 0.6 or unreliable ABI. All patients with diabetes in this study underwent digital subtraction arteriography and were diagnosed with non-reconstructable CLTI of the lower extremity. In addition, all patients received conservative treatment before SCS implantation.

Exclusion criteria were tumor or malignancy in the last 10 years, short life expectancy (<1 year), and no follow-up data. In addition, patients with rapidly progressive ischemia, necrosis of more than one toe, extensive infection, and/or extensive non-healing ischemic ulcers or gangrene were excluded from the study.

All patients with diabetes were discussed in a multidisciplinary conference, including vascular surgeons and interventional radiologists, and classified as inoperable and without options for revascularization. Reasons for non-alternative revascularization included occluded femoral vessels and distal lesions that could not be revascularized within the target artery pathway, lack of autologous venous material due to previous operations, lack of endovascular options, and inadequate response to conservative therapy.

Patient characteristics such as age, sex, nicotine use, hypertension, diabetes, coronary artery disease, ischemic rest pain (Fontaine stage III), or ulcer or necrosis (Fontaine stage IV) were obtained from clinical charts and surgical records. Patients with isolated resting extremity pain were diagnosed as Fontaine stage III, and patients with resting extremity pain and ulcers less than 3 cm in diameter were diagnosed with Fontaine stage IV. For better understanding across stages of disease complexity, all patients were classified according to the GLASS (Global Limb Anatomic Staging System) criteria. Operative data were collected during surgery using standardized data forms.

Wounds were graded using the Wagner scale and included if they measured ≥ 1 cm at initial assessment. This protocol is the standard extremity wound management protocol used by many specialized wound care centers. Chronic ulcers were defined as full-thickness ulcers that extended through the entire dermis into the subcutaneous tissue at least 6 weeks before starting therapy.

### 2.2 Follow-up and end points

Outcome data were assessed at 1, 6, and 12 months after implantation and annually thereafter for 10 years. These follow-up visits included a physical examination, technical inspection of the spinal cord stimulation, and documentation of pain claims. Patients who could not attend follow-up visits were contacted by telephone. The data of the included patients were collected until the last follow-up visit or death of the patient.

Ankle-brachial index (ABI) was measured whenever possible and performed in accredited vascular laboratories. Ankle and toe systolic pressures (mmHg) were measured using bilateral Doppler ultrasound in the posterior and anterior tibial arteries divided by the highest ipsilateral or contralateral brachial artery pressure. Generally, one pressure measurement was taken for each limb. ABI was considered unmeasurable if inflation of the blood pressure cuff to a pressure >250 mm Hg was insufficient to compress the arteries at the level of the ankle.

A visual analog scale (VAS) was used to measure perceived pain based on a continuum of values. The VAS is anchored between 0 (no pain at all) and 10 (severe pain). Patients also rated the skin temperature in the ischemic area using a VAS from 0 (maximum cold) to 10 (maximum hot) and were asked to complete a questionnaire about their satisfaction with the treatment and the World Health Organization Quality of Life – BREF (WHOQOL-BREF) was used. The WHOQOL-BREF is a self-report questionnaire that assesses four dimensions of quality of life (QOL): physical health, mental health, social relationships and environment. Items are scored from 1 to 5, and the raw area score is the sum of the respective item scores. All domain scores are then normalized to a range of 0–100.

Use of analgesics was recorded based on frequency of administration and type of analgesic. Opioids were classified as major analgesics, while NSAIDs were classified as minor analgesics.

The primary study endpoints were major amputation and all-cause mortality. Secondary outcomes were clinical outcome (Fontaine classification, pain perception, quality of life, surgical complications) and wound closure 1 year after initiation of therapy. Mortality was defined as death from all causes ≥1 year after intervention. Wound closure was defined as complete coverage of the epithelium.

Indications for amputation were progressive necrosis, uncontrolled pain or extensive infection and/or non-healing ulcers. Limb salvage was defined as no amputation. The extent of amputation was classified in order of increasing disability as absent (no amputation or only minor amputations of the lower leg), moderate (unilateral amputation below the knee), or major (at or above the knee level).

### 2.3 Surgical procedures

All procedures were performed under anesthesia control. The dorsal epidural space was punctured under local anesthesia and a thin multi-electrode wire was placed between the 10th–12th levels of the thoracic vertebra under x-ray control. Intraoperative paresthesia mapping confirmed coverage of ischemic areas (15). The lead was anchored to the supraspinal fascia. A subcutaneous pouch for a pulse generator (Genesis™ Implantable Pulse Generator, St. Jude Medical/Abbott, Plano, Texas, USA) was created in the left iliac fossa. A subcutaneous extension connected the lead and the pulse generator. Antibiotic treatment was performed prophylactically. All patients were usually discharged a few days after external telemetry programming of the pulse generator. The stimulation plan consisted of pulses repeated at a frequency of 50 Hz, a pulse width of 210/∼s, and an intensity (voltage) that produced a comfortable paresthesia in the ischemic area. One amplitude was programmed for the vertical position and the other amplitude for the horizontal positions.

Patients could start or stop stimulation, switch between two stimulation intensities, and were encouraged to use the stimulator as often as they wished. During follow-up visits, patients were questioned about stimulator function, emphasizing the adequacy of paresthesia in the ischemic area. Patients were advised to contact their treating physician between follow-up visits if paresthesia disappeared. Then the drug and/or stimulus was changed.

### 2.4 Statistical analysis

Collected data were statistically analyzed using GraphPad software version 9.5.0 (GraphPad Prism, San Diego, CA, USA). All data were analyzed retrospectively. Numerical variables are presented as mean ± standard deviation (SD). Two-sided student's *t*-test was used to determine the differences in continuous variables. Discrete and categorical variables are presented as percentages. Kaplan-Meier curves were used for the primary endpoint analysis of survival and amputation rates. A *P* value <0.05 was considered statistically significant.

## 3 Results

### 3.1 Patient characteristics

Between January 2010 and January 2017, 13 patients with diabetes were included in this small cohort analysis. A total of 5 (38.5%) patients were diagnosed with Fontaine stage III and 8 (61.5%) patients with Fontaine stage IV. The mean age for Fontaine stage III was 65 years (SD ± 7.94) and for Fontaine stage IV 61 years (SD ± 8.96). All patients were classified as GLASS stage III. GLASS stage was determined based on the scoring system reported previously in detail (16). Most patients were men (76.9%). The mean age ± standard deviation (age ± SD) of the included population was not significantly different between male and female patients. The long-term follow-up period was 10 years.

All patients were considered unsuitable for vascular reconstruction. Two patients (15.4%) had no autologous vein, 10 patients (76.9%) had no matching artery, and 1 patient (7.7%) had none. Angiographic evaluation showed that 9 patients (69.2%) had stenosis of more than 65% of the vessels or occlusion in the upper or lower leg, and 12 patients (92.3%) had at least one vessel occlusion and no suitable vessels for revascularisation.

Diabetes, hypertension, nicotine use, and coronary artery disease were the most common comorbidities, and every study patient had at least 2 of the listed conditions. The main clinical characteristics are summarized in Table 1.

**Table 1:** Baseline characteristics of patients (*n* = 13).

Variable	*n*	%
Sex		
Male	10	76,9
Female	3	23,1
Age		
Mean (years)	63,6	±8,8
Range (years)	48 to 74	
Critical limb ischemia classification		
Fontaine's stage III	5	38,5
Fontaine's stage IV	8	61,5
GLASS classification		
Stage I	0	0
Stage II	0	0
Stage II	13	100
Risk factors		
Diabetes	13	100
Hypertension	13	100
Nicotne use	12	92,3
Coronary artery disease	11	84,6
Previous treatment		
Mediacal therapy	13	100
Surgical revascularisation	11	84,6
Multiple attempts of endovascular treatment	13	100
Anticoalgulants		
Monotherapy	13	100
Two medications	9	69,2

During follow-up, there were no infections or other complications related to the procedure or the device. Of all cases, four (30.77%) permanent SCS devices were replaced due to battery life.

### 3.2 Survival and amputation rates

**Figure 1:**
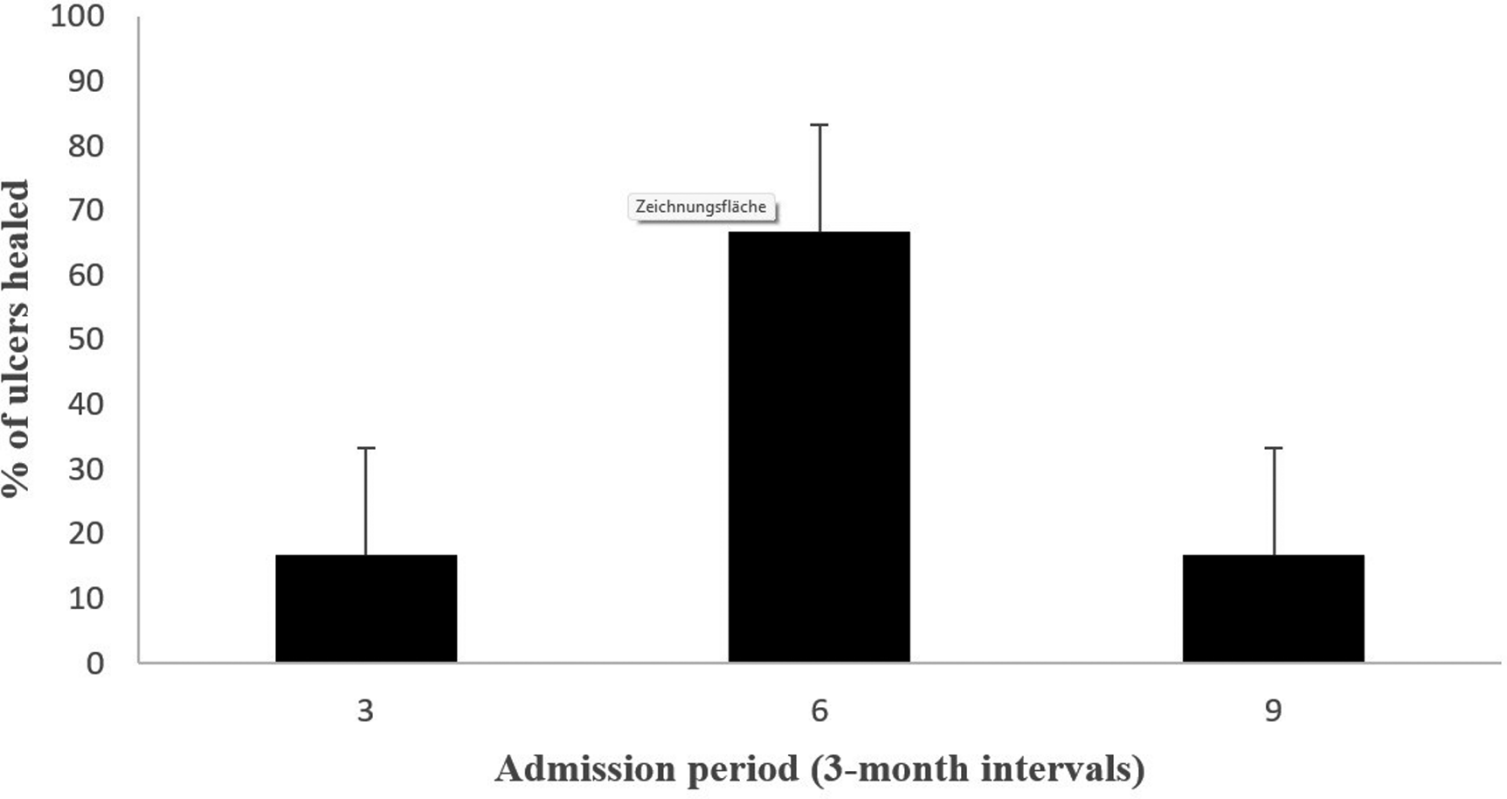
Healing process of ulcers after admission.

At 1-year follow-up, limb salvage was achieved in 92.3% of patients (12/13) and limb ulcers healed in 75% of patients (6/8), Figure 1. No patient died during the one-year follow-up. During long-term follow-up, a total of 4 patients (30.77%) were amputated at or above the knee (“major amputation”); all were Fontaine Stage IV. These patients were amputated at 6, 18, 42 and 48 months, and amputations were performed at disease progression. No mild or moderate amputations were performed. A comparison with a similar patient profile to our previous study, but with non-diabetic patients (*n* = 16), shows that patients who received the SCS device lived longer overall and had a significantly lower amputation profile (17).

**Figure 2:**
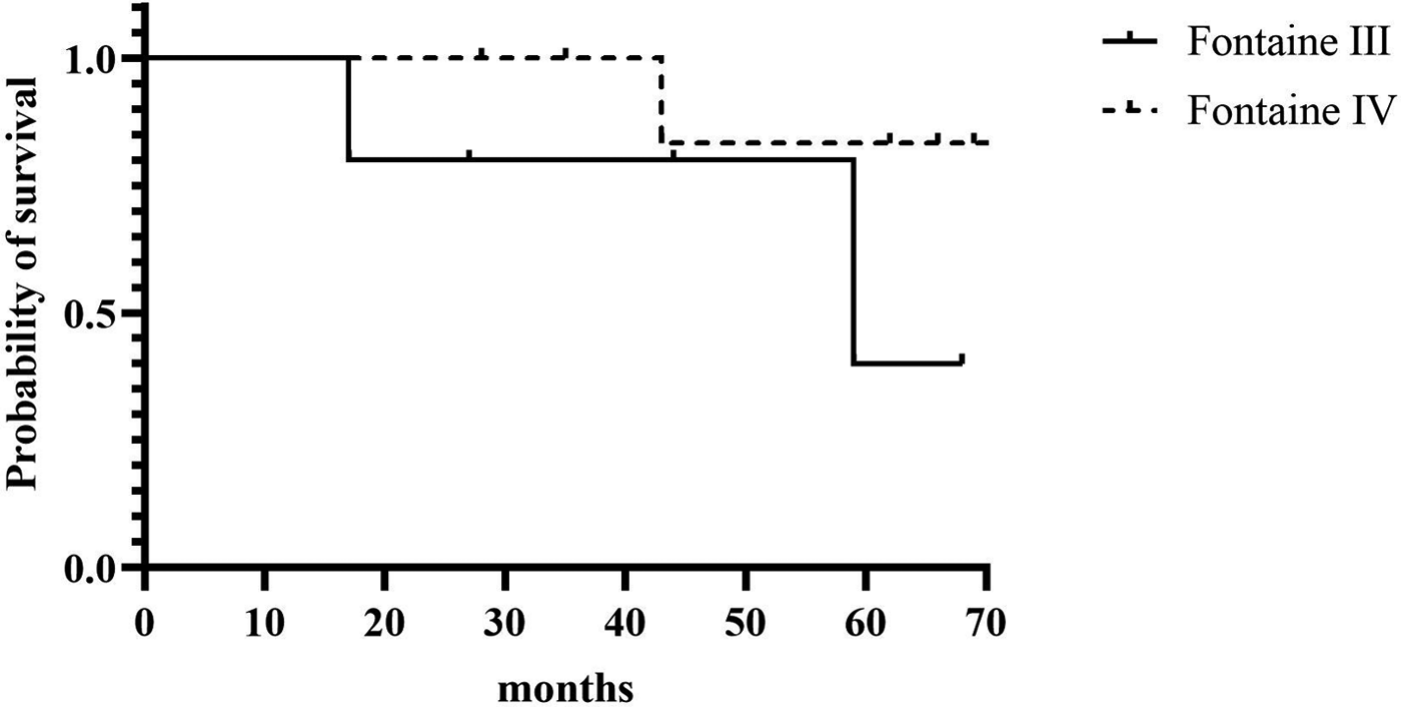
Kaplan-Meier overall survival for all patients and according to Fontaine stage III and stage IV.

**Figure 3:**
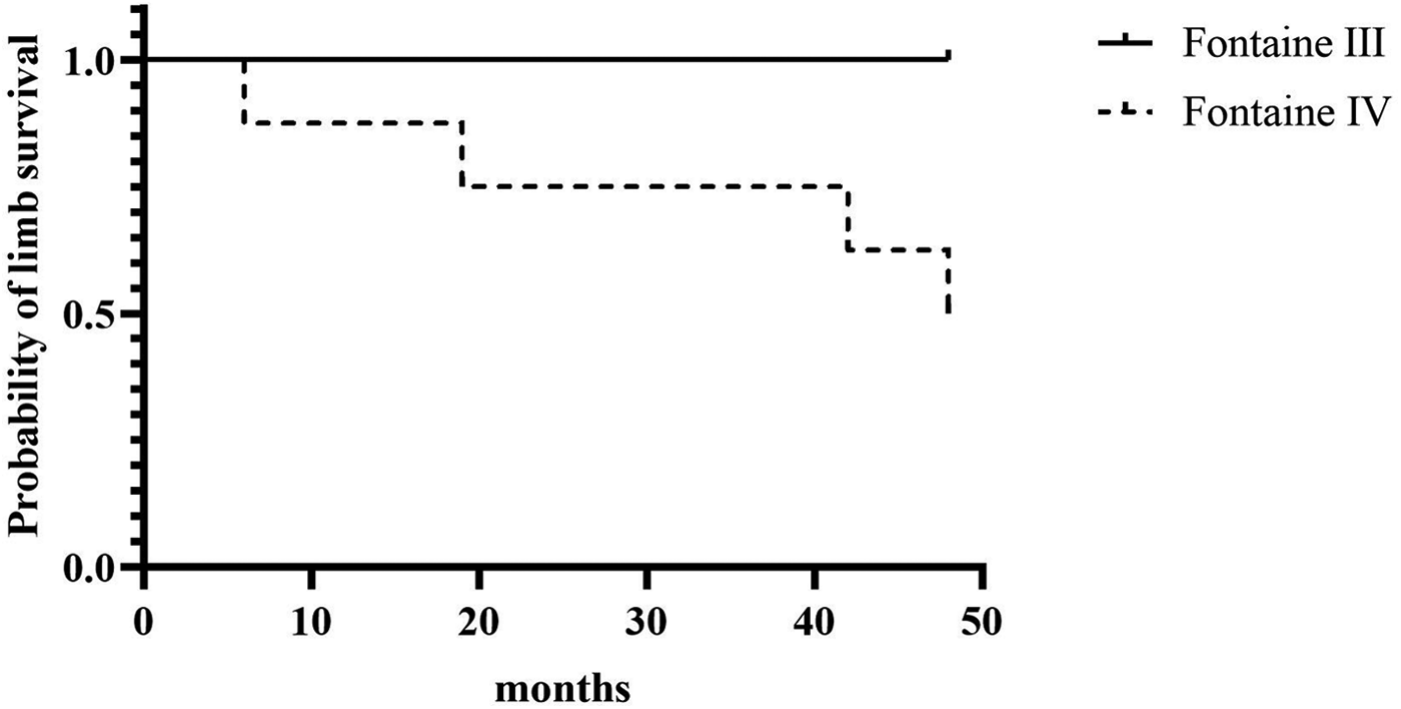
Kaplan-Meier amputation-free rate for all patients and according to Fontaine stage III and stage IV.

A total of 3 patients died (23.1%); one patient had previously a major amputation. Causes of death were cardiac (*n* = 2) and cancer (*n* = 1; bronchial), and time to death ranged from 17 to 68 months after SCS implantation. Kaplan-Meier overall and limb survival curves for all patients and Fontaine stages III and IV are shown in Figures 2, 3.

### 3.3 Pain score and skin temperature

**Figure 4:**
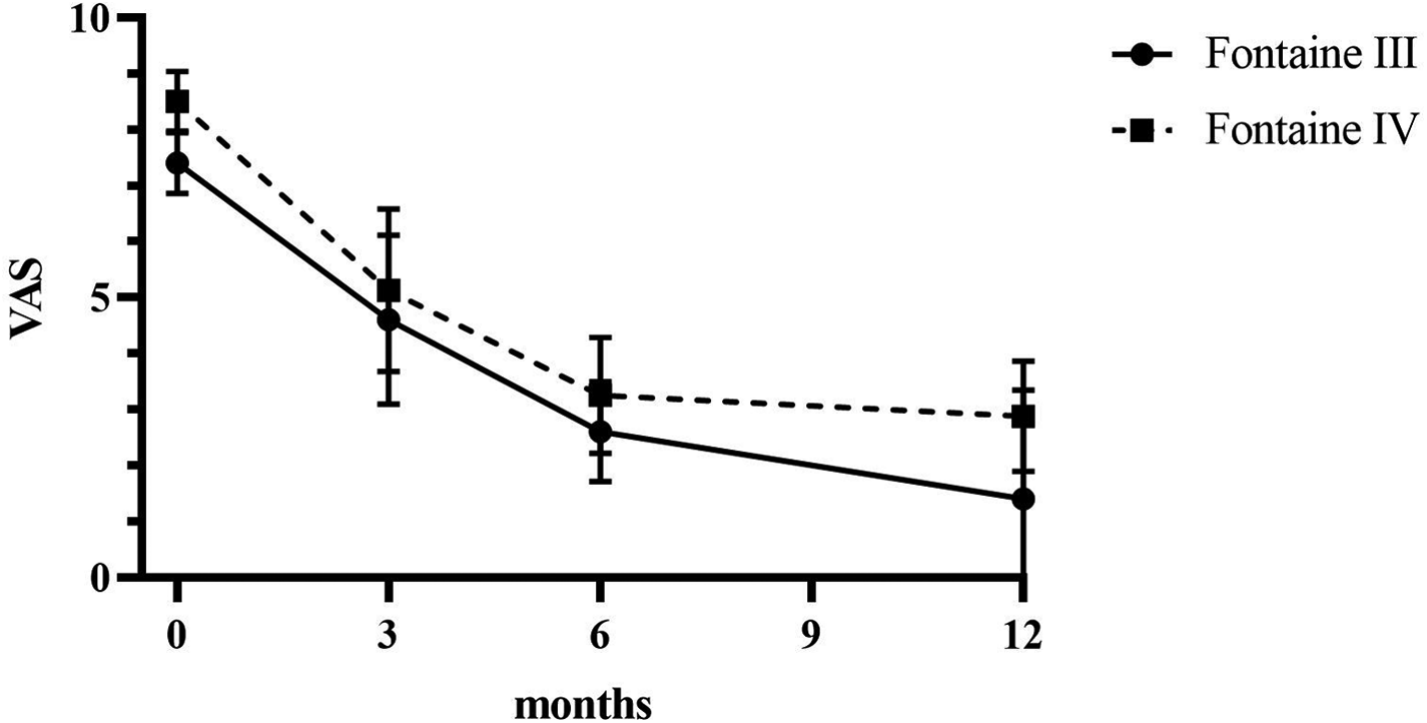
Intensity of ischemic pain assessed by using VAS before and after SCS implantation.

**Figure 5:**
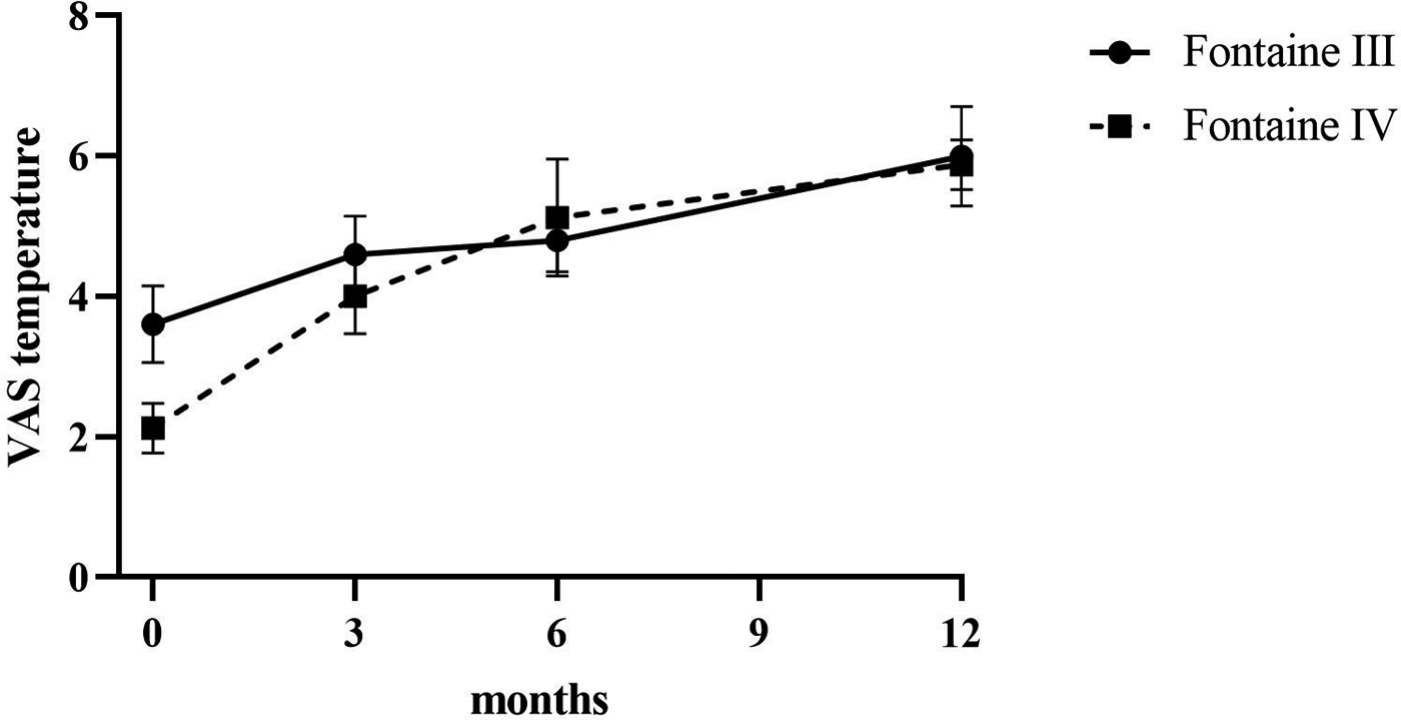
Skin temperature assessed by using VAS before and after SCS implantation.

**Figure 6:**
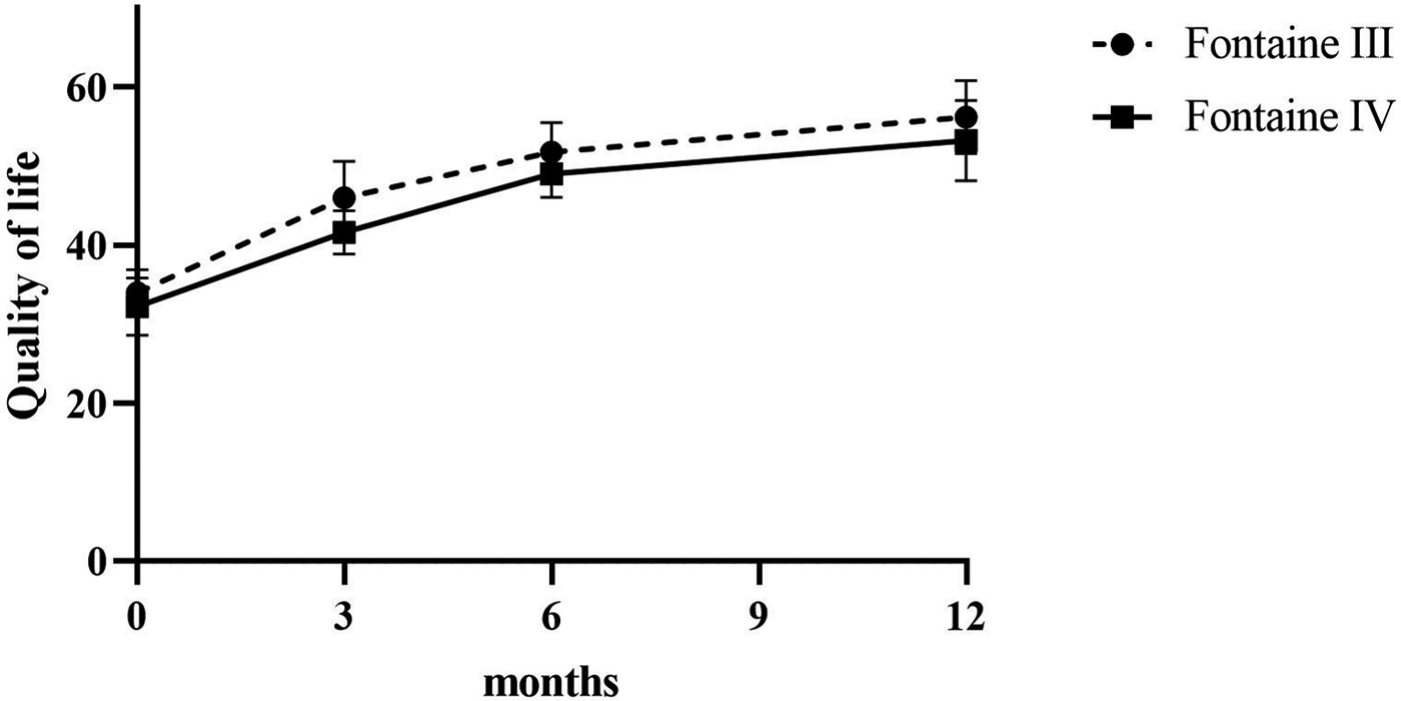
Quality of life before and after SCS implantation.

**Figure 7:**
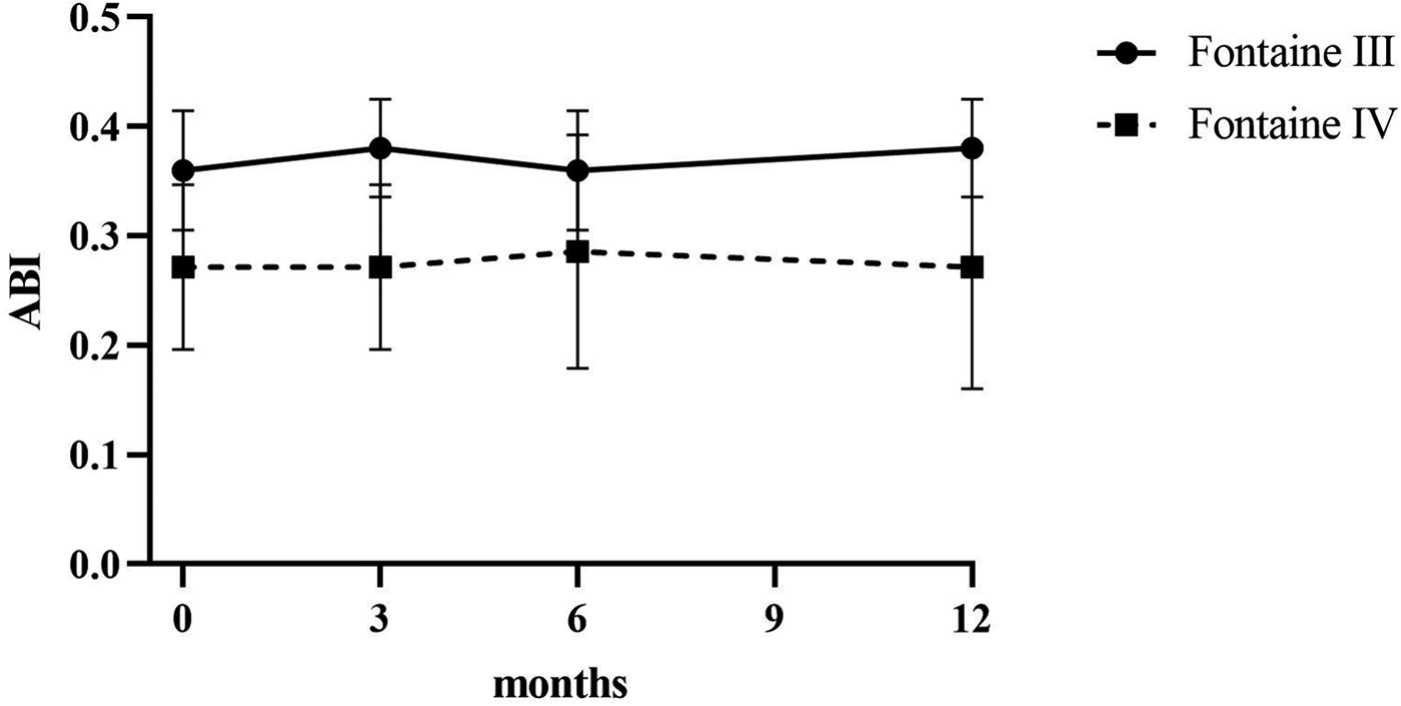
Ankle-brachial-Index (ABI) before and after SCS implantation.

Patients reported a significant reduction in pain intensity on the VAS from 7.80 ± 0.75 at baseline to 5.00 ± 1.10 at 3 months, to 2.60 ± 0.80 at 6 months, and to 3.50 ± 0.5 at 12 months (*p* < 0.05) in patients of Fontaine stage III. In patients of Fontaine stage IV, pain intensity on VAS decreased from 8.5 ± 0.50 at baseline to 5.13 ± 1.36 at 3 months, to 3.25 ± 0.97 at 6 months, and to 2.88 ± 0.93 at 12 months (*p* < 0.05). A similar improvement was observed in ischemic skin temperature and quality of life. During the 1-year follow-up period, VAS skin temperature more than doubled and quality of life scores were 56% higher in Fontaine stage III patients and 53% higher in Fontaine stage IV patients. No significant increase in ABI value was observed over time (Figures 4–7).

Patients reported complete pain relief (100%) at 24 months (VAS at 24 months = 0.00, *p* < 0.0001). Improvement in quality of life remained stable during the 48-month observation period.

### 3.4 Pain medication

Before SCS implantation, 5 patients regularly used major analgesics, 3 patients used minor analgesics, and 5 patients used a combination of both. Medications were administered daily. After 3 months, 2 patients were intermittently using major analgesics and 4 patients were using minor analgesics. After 12 months, only 4 patients used minor analgesics. During long-term follow-up, patients occasionally used minor analgesics and did not report major analgesic use.

## 4 Discussion

Revascularization is the primary treatment for diabetic patients with CLTI, but many are ineligible due to various reasons (extensive and diffuse, often infrapopliteal, atherosclerotic lesions, comorbidity, and/or lack of a suitable bypass graft) (6). Hence, non-invasive treatments are being explored to alleviate CLTI side effects, as medical treatment is mostly palliative.

Previous studies demonstrate the effectiveness of SCS in ischemic pain relief and in dramatically improved quality of life (12, 13, 17–23). The mechanism of action of SCS is still not fully understood and many theories have been reported. Generally, electrodes in the epidural space stimulate sensory unmyelinated c fibers and myelinated Aδ fibers, activating cell signaling molecules such as extracellular signal-regulated kinase (ERK) and protein kinase B (AKT), which stimulate a transient receptor potential vanilloid receptor 1 (TRPV1) (18, 19, 24–26). Activation of TRPV1 and depolarization of nerve terminals leads to the release of vasodilators such as calcitonin gene-related peptide (CGRP), which is a potent microvascular vasodilator. CGRP release is responsible for endothelial nitric oxide (NO) release and stimulates smooth muscle cell relaxation. These effects lead to a decrease in vascular resistance and an increase in local blood flow (18). In addition, SCS inhibits sympathetic vasoconstriction by inhibiting sympathetic nicotinic transmission at the ganglionic and postganglionic levels (18, 27, 28). Multiple mechanisms may be active simultaneously, with inhibition of autonomically mediated vasoconstriction and activation of vasoactive agents contributing to the efficacy of SCS (29).

The beneficial effects of SCS in diabetic patients with PAD include a reduction in resting pain and an increase in temperature (20, 29). The goal of SCS in patients with PAD diabetes is not only to achieve effective pain relief, but also to promote trophic-functional recovery of the body segment affected by the advanced ischemic process. In our study, all five diabetic patients with SCS Fontaine stage III had pain improvement at 3-month follow-up. In addition, most patients with Fontaine stage III reported no pain at one year after implantation and had a significantly higher quality of life than patients with Fontaine stage IV. These results suggest that treatment of CLTI with SCS in stage III-IV is beneficial rather sooner than later, although both groups showed progressive and significant improvement at 1-year follow-up. These results are consistent with contemporary literature reports showing the efficacy of SCS in patients with CLTI (17, 20–23, 30). Medical management of PAD patients with SCS according to microcirculatory parameters like TcPO2 is currently considered the standard of care. It has been shown to help reduce pain, stop injury progression and improve the chances of limb salvage (24, 26, 27). However, we did not perform microcirculatory assessments in this study, and no significant macrocirculatory effects were observed. Selection of the right candidate for SCS among patients with PAD is based on certain basic criteria outlined by Kumar et al. in their study (29). All of our diabetic patients who participated in SCS implantation had severe symptoms and did not respond or were not suitable for conventional surgical, endovascular and medical therapy. For revascularization treatment planning, the GLASS criteria offer an improvement over lesion-based systems. The staging system involves defining a preferred target artery path (TAP) and estimating limb-based patency (LBP) to categorize stages of complexity for intervention. GLASS stages I to III correspond to low-, intermediate-, and high-complexity infrainguinal disease patterns and are linked to immediate technical success and 1-year LBP for revascularisation procedures (16). Thus, GLASS stages provide a more objective assessment of infrainguinal disease complexity in the lower extremities. Our patients had a complex multilevel occlusive disease, were unsuitable for revascularization, and had no target artery provide continuous in-line flow to the ankle and foot. Whereas major amputation may be suitable for some of these patients, clearly a significant number might benefit from non-revascularization-based treatments such as SCS.

We report here a significant improvement in VAS pain score and quality of life and a reduction in pain medication when SCS treatment was established. The improvement observed three months after implantation was still present at long-term follow-up and was maintained at 10-year follow-up. There was a statistically significant reduction in pain intensity at all times. This is in accordance with Klinkova et al. who also reported 100% pain relief in SCS patients 12–14 months after implantation (17).

At 1-year follow-up, wound closure was achieved in 75% of patients in our series. Previous studies of limb loss in patients with extremity injuries have identified diabetes and renal failure as risk factors for amputation (31, 32). We did not identify these risk factors, but this may be related to the small number of patients. In our previous study, none of the non-diabetic patients required amputation (30). In this present series, amputation was required in four patients (30.77%); all were Fontaine stage IV patients. In general, SCS should not be used to treat patients with extensive gangrenous lesions of the legs (classified as Fontaine stage IVb). The recommended treatment for these patients is first-line amputation if they are refractory to medical therapy or vascular reconstruction.

CLTI is associated with both an increased risk of amputation and an increased risk of cardiovascular disease and mortality, which vary depending on the severity of CLTI. One-year amputation rates range from 15% to 20%, and 1-year mortality from 13.5% to 40% (33). The significant differences in amputation and death rates may be due in part to differences in definitions of CLTI in different studies. For further assessment of the natural history of CLTI, a recent meta-analysis reviewed studies that included only patients with untreated severe CLTI and found a 22% 1-year amputation rate and a 22% 1-year mortality rate (34). In our study group, three patients died (23.1%); four patients (30.77%) had a major amputation. Causes of death were cardiac (*n* = 2) and cancer (*n* = 1; bronchial). Based on previous studies, the 5-year mortality in diabetic patients with foot ulcers was generally in the range of 40% and increased to 63% in patients with limb ulcers that were subsequently amputated (35). In general, the coexistence of PAD and diabetes greatly complicates the management of these conditions. In addition, these diseases multiply the risk of amputation and poor survival.

SCS complication rates range from 3 to 17% and include hardware failure (lead migration, generator failure) and infection at the epidural lead or generator site (11, 36–38). Infections at the SCS electrode implantation site are more frequent when the device is implanted in two sessions, but immediate implantation can reduce this risk, as was done in our study. Our study found no complications related to the SCS procedure or device.

## 5 Limitations

This study acknowledges limitations such as small sample size, single center enrollment, retrospective analysis, and lack of TcPO2 measurements. A larger, more diverse population is necessary to verify these findings and avoid selection bias.

However, SCS- was proven to be safe and effective for diabetic patients with PAD- suffering from ischemic pain and tissue loss. SCS was able to control pain and enhance quality of life over time. In all patients, initial improvements were maintained for up to ten years. Clear patient selection criteria are crucial for treatment success, and patients must be unable to undergo drug therapy or revascularization.

When evaluating a novel treatment, it is important to consider the natural progression of the disease and the potential for a placebo effect. It is well known that intermittent claudication tends to improve over time, but it usually occurs over months, not days, and usually early in the course of the disease. The usual clinical course of patients with severe persistent rest pain is to remain stable or worsen (39, 40).

## 6 Conclusions

The study highlights the positive effects of SCS in diabetic patients with PAD. SCS not only reduces resting pain but also promotes healing in the body segment affected by advanced ischemia. We suggest that SCS may play a role in the treatment of advanced arterial disease in diabetic patients when all other therapies have failed. Early intervention with SCS in stages III-IV of PAD seems beneficial for long-term outcomes. However, further research is required to determine the specific benefits of SCS and its impact on vascular health in diabetic patients with critical limb ischemia.

## Data Availability

The raw data supporting the conclusions of this article will be made available by the authors, without undue reservation.
